# A compact, lightweight, variable-energy cyclotron for conventional and FLASH ion beam radiotherapy

**DOI:** 10.3389/fonc.2025.1648237

**Published:** 2025-10-21

**Authors:** D. Winklehner, J. V. Minervini, L. Bromberg, E. Forton, J. Mandrillon, P. C. Michael, A. Radovinsky

**Affiliations:** ^1^ Department of Physics, Massachusetts Institute of Technology, Cambridge, MA, United States; ^2^ Plasma Science and Fusion Center, Massachusetts Institute of Technology, Cambridge, MA, United States; ^3^ Ion Beam Applications SA, Louvain-la-Neuve, Belgium

**Keywords:** radiotherapy, cyclotron, superconductivity, FLASH radiotherapy, particle accelerators

## Abstract

**Introduction:**

The advantage of ion beam radiotherapy for cancer lies in its low dose proximal and distal to the tumor, owed to an energy-dependent depth-dose profile, the Bragg-peak. However, conventional techniques to achieve different energies often rely on degraders, which compromise the quality and intensity of the beam and produce secondary radiation.

**Methods:**

We propose a novel conceptual design for a compact accelerator capable of delivering ion beams (e.g., protons or carbon ions) with variable energy from 70 to 230 MeV/amu. Removing all magnetic iron from the device yields a linear relationship between coil current and cyclotron magnetic field, and, thus, smooth scaling of the output beam energy. We base our findings on finite elements calculations, particle ray-tracing and particle-in-cell simulations.

**Results:**

In the absence of magnetic iron, we achieve a much lighter system with improved magnetic shielding and significantly reduced secondary radiation that can provide ion beams at variable energy while providing the high beam intensity necessary for the promising FLASH technique at all output energies.

**Discussion:**

This design represents a promising advancement in ion beam radiotherapy, combining energy flexibility, reduced radiation hazards, and compatibility with high-intensity techniques. It may pave the way for more efficient, compact, and clinically versatile accelerator systems.

## Introduction

**Figure 1:**
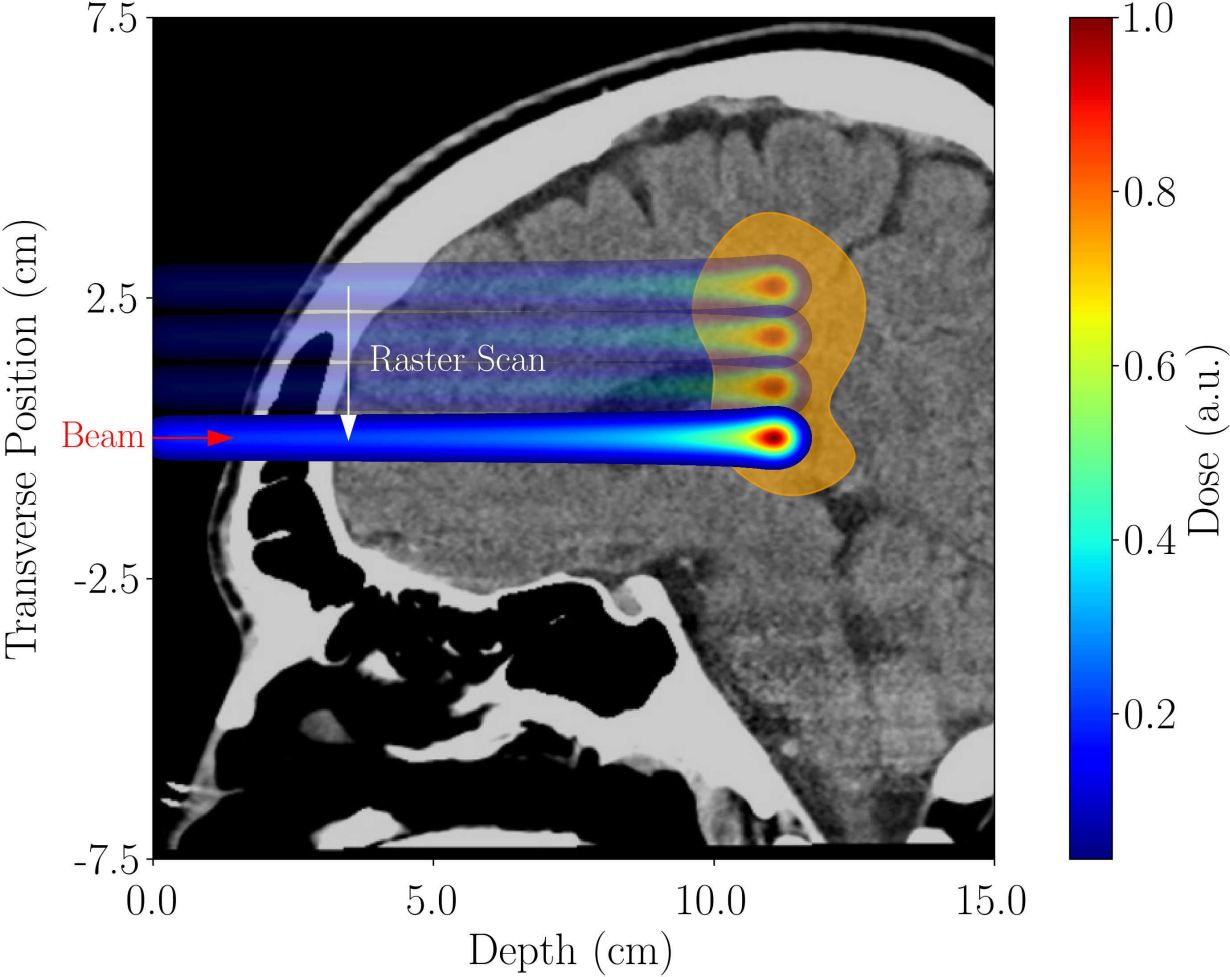
Rastering across a brain tumor using a pencil beam. The energy-dependent depth-dose profile (Bragg peak) leads to the sparing of healthy tissue distal and proximal to the tumor location.

Hadron and ion beam radiotherapy treatment of cancer cells is currently the most advanced form of radiation therapy (1–3). The advantage of ion beam therapy lies in the high spatial conformity of delivered dose with tumor volume due to the low dose proximal and distal to the tumor, owing to an energy-dependent depth-dose profile, the Bragg-peak (4) (see **Figure 1**), sparing healthy tissue. Proton Beam Radiotherapy (PBRT) is the most common form of hadron therapy. Carbon beams offer some treatment advantages over PBRT, but the facilities are much larger and more expensive. There are thus fewer of them, with limited availability. Another novel method for delivering the radiation dose, FLASH therapy (5–9), has received considerable attention lately. The basic idea behind FLASH is that a significant increase in dose rate (from below 1 Gy/s to over 100 Gy/s) during treatment fundamentally impacts the tissue response to the radiation. The exact chemical and biological effects are still to be understood (10, 11). Still, there is a strong indication that FLASH can significantly improve treatment quality (faster tumor response, significant sparing of healthy tissue) and dramatically change the course of cancer treatment. The higher doses also lead to a much smaller number of treatment sessions (hypofractionation), which improves patient throughput and reduces costs. Combining the current superiority of hadron therapy with potential FLASH delivery can further enhance the benefit to the patient.

Safely treating patients at much higher dose rates is a significant technological challenge for existing proton therapy systems. Indeed, present medical synchrotrons do not provide the required dose rate for FLASH, especially at low beam energy (12, 13). On the other hand, cyclotron-based commercial proton therapy systems have achieved FLASH dose rates, but only in research mode and at limited energies (12–14). Cyclotrons, therefore, constitute a good option for FLASH therapy if we can make them variable-energy with no significant loss in beam current at lower energies.

In recent years, superconductivity has made it possible to reduce the weight of cyclotrons by an order of magnitude and their dimensions by a factor of three to four (15, 16). However, they still weigh 20–100 metric tons and provide only a single fixed output beam energy. Reducing the beam energy from a conventional cyclotron is usually done using an energy degrader, a device made out of graphite or beryllium. Its variable thickness intercepts the beam to adjust its energy to match the tumor depth. This energy degrader enlarges both the spatial beam size and its energy spread, so the transmission efficiency through the beamline also deteriorates. At low beam energies, the degrader and the resultant need for beam collimation reduce the beam intensity to such an extent (down to< 1% at 70 MeV) that it cannot be used for FLASH treatment (17, 18). Furthermore, the degrader is a source of secondary radiation, requiring additional shielding and adding system complexity. In short, today’s cyclotron-based systems can achieve the beam intensities required for FLASH at their maximal energy but not at low energies. In conventional pencil beam scanning, low-intensity irradiation of tumor layers closer to the surface is achieved by degrading high-intensity, high-energy beams. The significant current losses (up to >99%) principally define the necessary shielding around the system, notably impacting cost and footprint. If the degrader can be omitted, the accelerated current in a pencil beam scanning system can be much lower, reducing activation of some accelerator subsystems and the neutron heating of the cold mass in the case of SC cyclotrons, which typically have an extraction efficiency between 35% and 50% of the accelerated beam (16).

Here, we present an advanced synchrocyclotron design concept that uses superconducting coils only. Eliminating the iron yoke reduces the cyclotron’s weight by another factor of four over the lightest existing systems (19–22). The idea of omitting the iron yoke was first presented for isochronous cyclotrons at conferences from 1984 to 1988 (23–26). However, these were either purely theoretical, or iron was still used to shape the azimuthally varying field (AVF), making smooth energy scaling impossible. No beam dynamics studies were presented. Replacing the poles in an AVF cyclotron with coils was also presented by Dey et al. (27) for a cyclotron accelerating up to 30 MeV. The use of shims and iron for magnetic shielding was retained in that study.

We propose to go fully iron-free, which negates any saturation effects and yields a linear relationship between coil current and cyclotron magnetic field, and, by extension, the beam momentum, thus allowing a smooth variation of the cyclotron beam energy. Most importantly, the smooth variation of the beam energy with the coil current results in the same beam intensity and focusing properties at any output beam energy, from 70 MeV to 230 MeV (protons). Another advantage of going iron-free is the lower inductance, enabling faster coil current changes. We discuss the physics and technical aspects, e.g., adjusting the RF frequency of the accelerating electric fields to match the beam particles’ revolution frequency at all times. Our design removes the beam intensity restrictions at low energies, enabling FLASH throughout the entire treatment energy range. An alternative to pencil beam scanning is the generation of a conformal dose distribution using a passive range modulator (28). This can be done in a conventional setup as well as in FLASH scenarios. For conformal FLASH (29, 30), setting the maximum beam energy to reach only to the distal edge of the tumor preserves ion beam radiotherapy’s ability to spare surrounding healthy tissue during treatment, avoiding the use of a higher energy range shifter, which would aggravate the distal falloff of the beam behind the tumor, while also avoiding the additional neutron dose produced in this shifter. In our design study, we assumed an average extracted beam current of 500 nA (0.6% duty cycle at 1kHz) to accommodate conformal FLASH.

Beyond the design we present for proton beams, the iron-free cyclotron can be adapted to carbon beams, as the authors have demonstrated conceptually elsewhere (19). The number of centers that can use carbon ions, though rising, is still small. Typically, they are synchrotron-based [e.g., MedAustron (31, 32)]. Our cyclotron would replace the much larger synchrotron, which could reduce the size of the entire carbon radiotherapy facility by about a factor of two, accompanied by a significant cost reduction.

## Materials and methods

### Requirements for smooth energy scaling

When E. O. Lawrence invented the cyclotron, he reportedly stormed into his graduate student J. J. Brady’s office, yelling, “R cancels R!” (33). He meant that the formulas describing beam dynamics in a classical cyclotron simplify so that the radius cancels. – It was a compelling observation enabling cyclotrons to accelerate charged particles to high energy, repeatedly using the same oscillating electric field. At the beginning of this project, we had a similar Eureka moment when we realized that removing all iron from the system would yield a linear relationship between coil current and magnetic field everywhere in the machine.

The magnetic field serves two purposes: To guide the bunches of particles on spiral orbits, with a revolution frequency corresponding to the oscillation of the accelerating radiofrequency (RF) electromagnetic field (created by an RF cavity, or “dee”); and to focus the beam radially and vertically. Radial focusing happens automatically in a dipole magnetic field. In a synchrocyclotron, we use so-called *weak focusing* for the vertical direction, which requires the magnetic field to decrease radially. To first order, the radial (r) and vertical (z) focusing is governed by the following equations (34):


(1) $$\begin{array}{ccc} (\text{a})\;n\left(r\right)=-\frac{\text{d}B}{\text{d}r}\frac{r}{B} & (\text{b})\;\nu_{r}=\sqrt{1-n} & (\text{c})\;\nu_{z}=\sqrt{n} \end{array}$$

Here, n is the field index, ν_r_ the radial tune, and ν_z_ the vertical tune. These tunes correspond to the number of oscillations each particle performs around the equilibrium orbit in the respective direction during one turn. Assume the “base design,” which delivers the highest desired particle energy $T_{0}$ , has a rotationally symmetric field $B_{0}\left(r\right)$ . The “magnetic rigidity” is then defined as


(2) $$B_{0}\left(r\right)r=\frac{p}{q}=\frac{m_{0}c}{q}\sqrt{\gamma^{2}\left(r\right)-1}$$

With $m_{o}$ the rest mass of the accelerated particle, c the speed of light in vacuum, q the charge of the particle and $\gamma \left(r\right)=1/\sqrt{1-v{\left(r\right)}^{2}/c^{2}}$ the relativistic gamma at each radius. If we desire a scaled-down energy $\alpha T_{0}$ ( α≤1 ) at the extraction radius R , using (2) and $\gamma \left(R\right)=1+\frac{T_{0}}{E_{0}}$ , where $E_{0}=m_{0}c^{2}$ , we can calculate a scaling factor η(α) for the magnetic field:


(3) $$\eta \left(\alpha \right)=\frac{B_{\alpha }\left(R\right)}{B_{0}\left(R\right)}=\sqrt{\frac{2\alpha E_{0}+\alpha^{2}T_{0}}{2E_{0}+T_{0}}}$$

Thus, if the scaling factor holds for each radius, we have


(4) $$n_{\alpha }\left(r\right)=-\frac{\text{d}B_{\alpha }\left(r\right)}{\text{d}r}\frac{r}{B_{\alpha }\left(r\right)}=-\frac{\text{d}B_{0}\left(r\right)}{\text{d}r}\frac{r}{B_{0}\left(r\right)}\;$$

and all transverse (r and z) focusing properties remain the same even for reduced final energy.

Magnetic fields in the presence of saturated iron do not scale linearly with the current driving the coils. In particular, local distortions will yield different beam dynamics in the distinct regions of the cyclotron for the various desired final energies. Hence, only an iron-free design lends itself to smooth energy scaling. However, the magnetic field is not the only crucial aspect of this design. The key to smooth energy scaling is controlling all fundamental acceleration parameters during the whole process and maintaining beam stability for any given output energy. Beyond the radial profile of the magnetic field, the critical control parameters are the RF voltage, -phase, and -frequency and how they change in time as particles are accelerated. In the longitudinal direction, particles perform *synchrotron oscillations* around the *synchronous particle* according to the principle of *phase stability* (35). This principle states that in phase-energy space, there is a closed contour and particles within will be stably accelerated, whereas particles outside will be lost in the machine, which would be prohibitive to dose control. Thus, particle phase and energy need to be carefully controlled. As described in **Appendix A**, synchrocyclotrons require the RF frequency to be lowered as particles gain energy and be brought back up to the starting frequency before the next set of bunches can be accelerated. The acceleration cycle repeats at a frequency of 1 kHz. Moreover, each final energy requires a unique frequency range. We use a ferrite tuner together with a high-bandwidth solid-state RF amplifier to gain fine control over the RF frequency, which we describe in the next section.

### The iron-free cyclotron

From the ion source to the extraction system, the main parts of the cyclotron are as follows: A central region with a Penning Ionization Gauge (PIG) ion source (36) and carefully designed electrodes to capture the particle bunches; the main acceleration region where particles are guided by a magnetic field, created by superconducting coils that are placed inside a cryostat, and are accelerated using a single dee; and the extraction region with resonant extraction, created by a separate set of coils. We will first discuss the main field and cryostat, followed by the central region and ion source, and conclude with extraction from the cyclotron.

**Figure 2:**
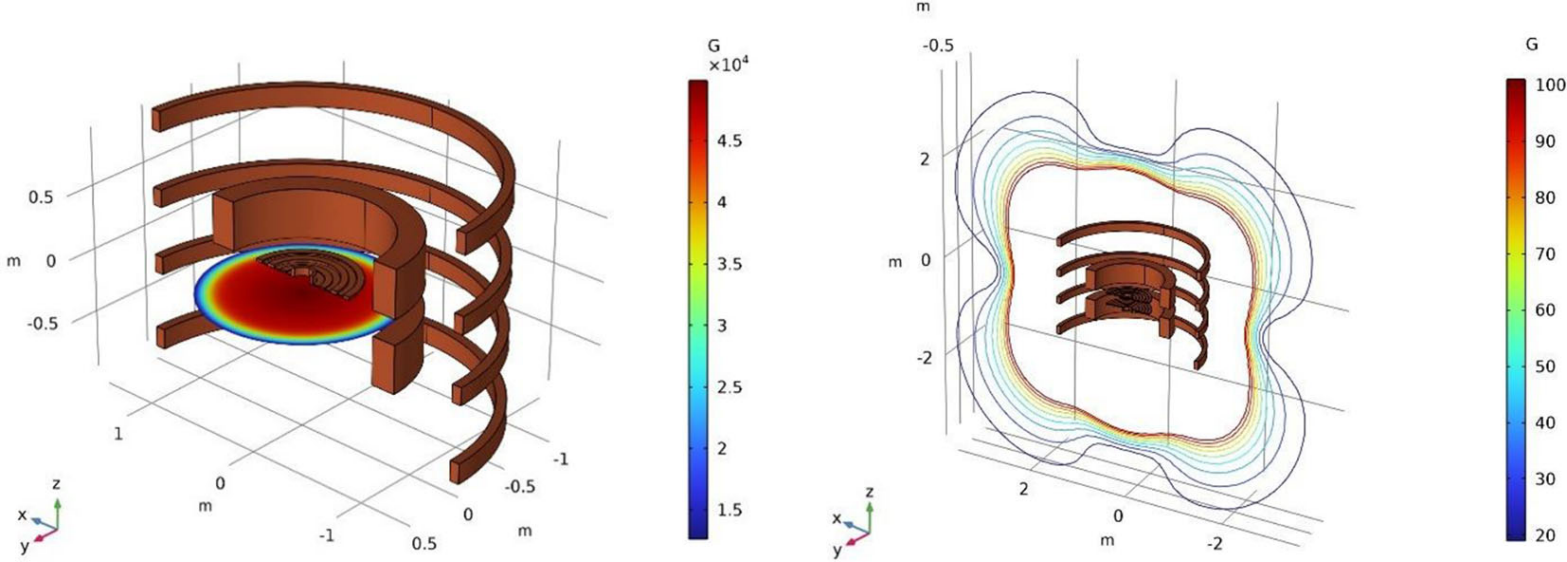
The superconducting coil assembly. From the inside out: shaping coils, main coils, compensation coils. Left: Main magnetic field with a peak B_z_ of about 50 kG in the center. Right: The stray fields around the cyclotron. Due to the active compensation, these are lower than the typical stray fields from a cyclotron with iron yoke, where the iron self-shields.

A set of primary coils creates the base magnetic field. The absence of magnetic field-shaping iron poles necessitates the addition of magnetic field-shaping coils to generate the precise radial variation of the axial field in the gap that is required to achieve beam stability. Similarly, the stray fields that would otherwise be contained in the iron yoke must be canceled using current-reversed magnetic shielding coils similar to those used in MRI magnets. We show the entire coil assembly with the corresponding magnetic fields at full power in **Figure 2**. In a synchrocyclotron, the magnetic field is symmetric with respect to rotation about the z-axis (vertical). The field strength decreases with radius to achieve vertical focusing, which keeps the beam from drifting apart vertically. The maximum field is determined by the desired extraction radius, particle energy at extraction, and particle mass according to Equation 2.

### The superconducting coils

The required magnetic field profile in the acceleration gap is created by superconducting coils. Thus, the size of the accelerator is no longer dependent on the magnetic saturation limit of iron (~2 T), allowing a very compact design by increasing the magnetic field generated by the superconducting coils to unprecedentedly high values, limited only by the choice of type of superconductor and engineering design limits. For example, the peak field in the gap is limited to about 6 T using niobium-titanium (NbTi) superconductor and about 12 T-15 T if Nb_3_Sn is used. One can even consider using a High-Temperature Superconductor (HTS) from which solenoid magnets have been built that are now operating at fields up to 32 T with paths up to 40 T (37), although the realistic limit is much lower due to other practical physics and engineering constraints. We provide more details on the use of HTS for the peeler-regenerator system as part of the extraction system below.

**Figure 3:**
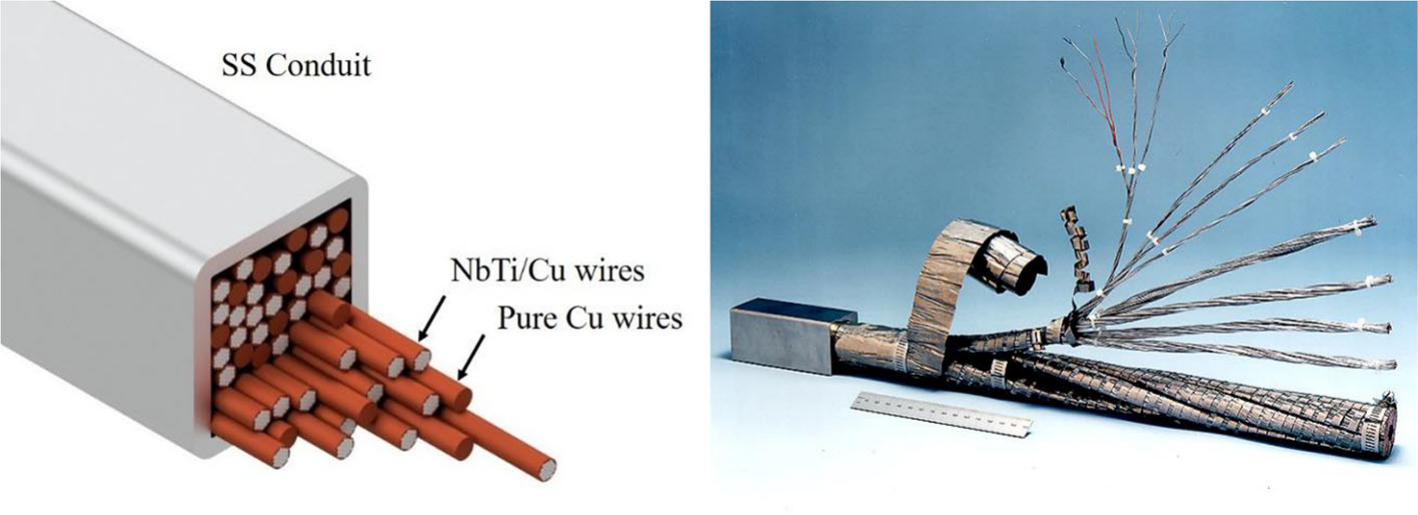
Left: Schematic of a cable-in-conduit conductor showing multiple pure copper wires mixed with copper-enclosed NbTi filaments, all inside a stainless steel (SS) conduit. Right: Photograph of a CICC.

In our design, the coils are made of Cable-In-Conduit Conductors (CICC) (38) with multifilamentary composite superconducting strands made of NbTi filaments in a copper matrix, as well as additional pure copper wires (see **Figure 3**). All wires are enclosed in a stainless-steel conduit, which is sealed after the void between wires is filled with high-pressure helium at room temperature (39, 40). As the magnet is cooled from room temperature to 4.5 K, the single-phase, high-pressure gas becomes a supercritical fluid that acts as a heat sink for AC losses in the superconductor. Circuit A, containing the main and shielding coils connected in series, carries a maximum operating current I_op_ = 3 kA. Circuit B, powering the field shaping coils, carries I_op_ = 0.5 kA. The necessity to cool the lead wires that carry current from room temperature to the superconducting magnets imposes the largest heat load on the cryogenic circuit. The combined 3.5 kA coil current requires about 300 W cooling capacity at roughly 68 K (41) to cool the upper stage of the current leads. This is readily provided by a single Gifford-McMahon or pulse tube type cryocooler, such as the Cryomech AL630. The maximum current corresponds to a peak field of B_z, in_ = 4.98 T in the center of the machine, and B_z, ex_ = 4.64 T at the 50 cm extraction radius, to generate a proton beam with final energy of 230 MeV.

### Cold mass structural support and cryostat

**Figure 4:**
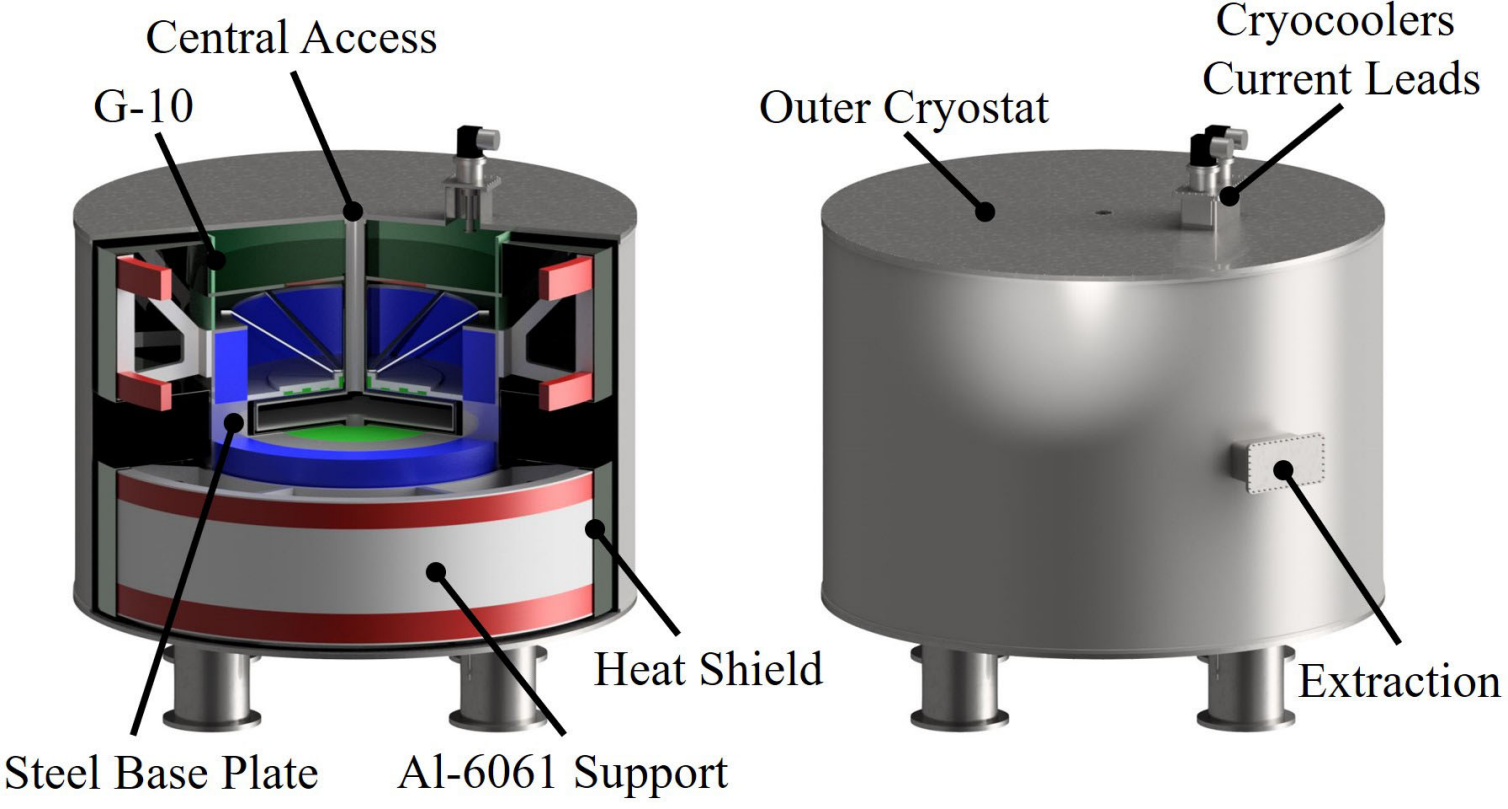
3D-rendered schematic of the cold mass and cryostat assembly. The coils are colored to guide the eye (blue – main coils, green – shaping coils, red – shielding coils). For clarity, the cassette with acceleration and extraction system is not shown.

Elimination of iron from the design also eliminates the electromagnetic forces that would otherwise be present between the cryogenic magnet cold mass and room temperature iron yoke, significantly simplifying the cold mass structural supports, while minimizing cryogenic heat loads. The coils are supported by a 316 stainless-steel base plate and Al-6061-T6 aluminum struts (see **Figure 4**). Structural analysis of the support structure and coils has shown that in our present design, all stresses and deformations are within tolerable limits.

**Figure 5:**
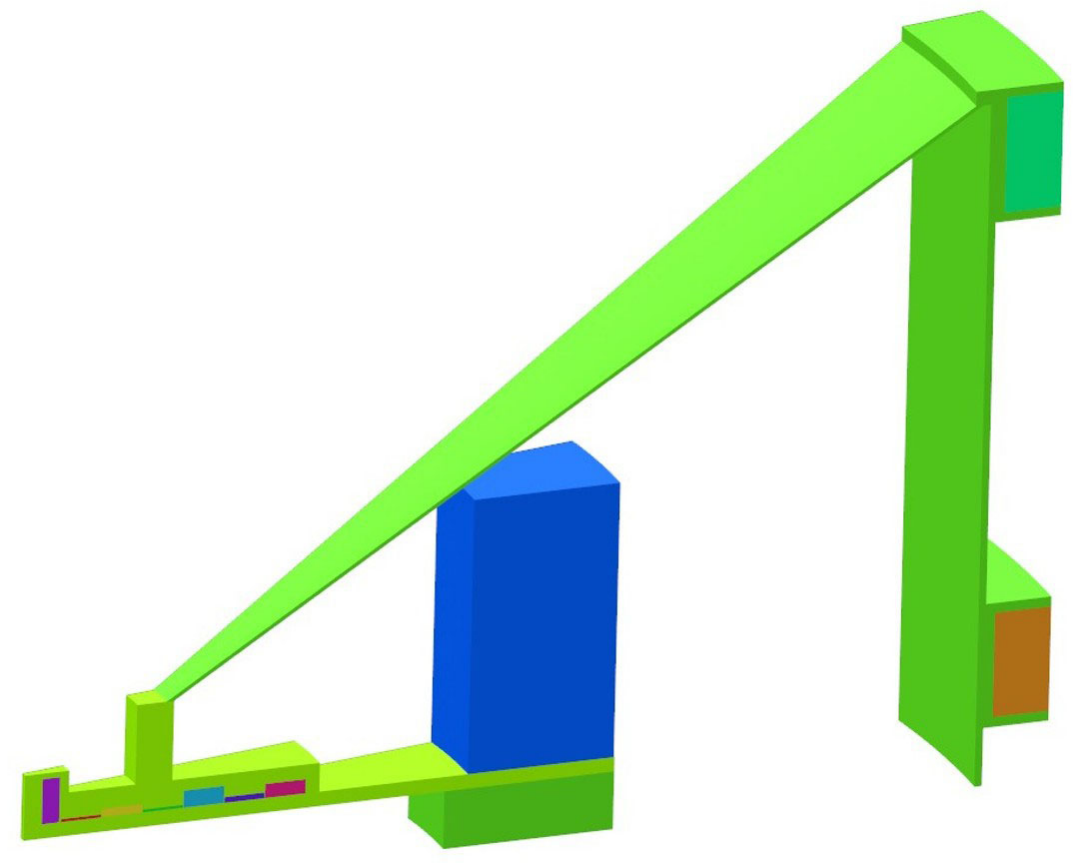
The axisymmetric structural model we analyzed using OPERA.

Structural assessment of our reference design was iteratively performed using the axisymmetric Opera model shown in **Figure 5**, with any deficiencies corrected by suitable modification to the design before the next iteration. The model used 1/36 rotational symmetry with a reflective symmetry about the cyclotron mid-plane. The windings were modeled as isotropic elastic media with the equivalent elastic moduli scaled from the conductor’s structural, stainless-steel conduit by the ratio of the conduit cross-sectional area to the overall insulated conductor area. This conservatively presupposes that the electromagnetic loads on the coils are supported only by the conductor conduit. The coil system is supported by a cold mass (CM) structure made chiefly of Al-6061-T6, except for the SS 316 structural base plate supporting the main and the field shaping coils. The field shaping coils are embedded in the base plate. The main coil contacts the base plate but is free at both at inner and outer diameter; the shielding coils are supported by a 2 cm-thick aluminum channel at their outer diameter and top and bottom surfaces. This provides structural support as well as a thermal conduction path to the cryocooler.

For the final design iteration, the stresses in the conductor conduit and in the cold mass support structure were well within those allowed by the ASME code for welded structural materials. The maximum hoop strain in the magnet windings were less than 0.1%, which is also well within an acceptable range for a helium cooled superconducting magnet system. The axial displacements of the energized windings were less than 1 mm in the model, well within the clearances permitted by the cryostat design.

The coils, structural support, and cryostat together weigh less than 14 tons, significantly lower than the lightest weight existing state-of-the-art machines. This is owed to the absence of iron and a lightweight construction comprising aluminum support struts, a heat shield, and G-10 spacers, as indicated in **Figure 4**. The cryostat volume is evacuated to 10–^5^ Torr for thermal insulation. The magnet cryostat employs features common to those found in superconducting rotating machinery, such as the use of segmented thermal radiation shields to minimize eddy current heating, to further reduce the heat loads on the 68 K cryogenic circuit. As before, the dominant heat load (roughly 1.25 W) on the 4K cold mass is produced in the HTS current lead connection between the 68 K thermal station and the magnet assembly; this can easily be accommodated by a pair of 1.5W @ 4K, two-stage cryocoolers for redundancy, such as the Sumitomo RTD-415D2, whose first stage also cools the cryostat’s thermal radiation shield.

### Magnetic field scanning

A power system design study was implemented to examine the suitability of our cyclotron concept for conventional 3D scanning, which requires rapid variation in the beam energy (i.e., magnet current) to sweep through the tumor depth (19, 20). For the study we assumed a worst-case scenario whereby we would need to sweep through the full 70 to 230 MeV energy range in 4.5 MeV increments and back again twice, that is, four times scanning of the tumor depth. By comparison a standard treatment plan typically requires only a half to a third of the scanning range assumed here.

To keep the total treatment duration to below 2.5 minutes a half second was allocated for each energy change and a half second was allocated for 2D scanning at that energy level. The study concluded that this mode of scanning was technically feasible but that it would require a power supply capable of up to 1.4 MW output power at roughly 730 V peak voltage. This type of power supply exists in the particle accelerator community and incorporates internal, regenerative capacitive energy storage to minimize potential disruption to the power supply grid (42, 43).

The estimated energy dissipation in the superconducting coil due to magnetization hysteresis (44) is below 5 mJ/cm^3^ for the assumed full treatment cycle, resulting in a temperature rise of only a few mK (20). This energy is stored temporarily in the helium stabilizer in the sealed conductor during treatment and easily removed by the cryogenic cooling system during the 10~12-minute dwell time between treatment cycles.

By comparison, the ability to operate at a single, fixed output beam energy during FLASH significantly reduces the demands on both the magnet power supply for fast current ramping, and on the cryogenic system for managing pulsed field losses. For FLASH 10 Gy or more must be delivered at 100 Gy/s or more, meaning that typical flash irradiation duration is in the order of few hundred milliseconds; those levels can be reached with this strategy: setting the energy once for the most distal layer in the tumor and using a ridge filter to avoid scanning between layers (29, 30).

### The RF system

We give special consideration to the radiofrequency (RF) system, providing the accelerating force acting on the beam. In the synchrocyclotron, the frequency must decrease with time as the particles are accelerated to higher energies to compensate for the relativistic increase of inertia (see Equation 2). Consequently, after a short train of particle bunches has been accelerated and extracted, the process must begin anew with the frequency returning to the starting point and a new set of bunches injected from the ion source in the center of the cyclotron. Three exemplary sets of parameters for different final beam energies are listed in **Table 1**. We propose a state-of-the-art technique to accommodate this acceleration profile and the non-linear change of frequency with time during each cycle by using a solid-state, biased-ferrite tuner (45) combined with a wide-band RF amplifier and a computer-programmable Low-Level RF (LLRF) oscillator to allow agile frequency and voltage control, while still achieving the required pulse repetition rate of 1 kHz. This tuner is coupled with the RF cavity and adjusts its resonant frequency to match the beam revolution frequency in the magnetic field at all times.

**Table 1:** Relevant parameters for three exemplary final beam energies used in simulations.

Final energy (MeV)	70	150	230
B_center_ (T)	2.64	3.94	4.98
B_extraction_ [T]	2.44	3.64	4.6
f_RF, start_ (MHz)	40.26	60.13	75.91
f_RF, end_ (MHz)	34.58	47.86	56.28
Turns	~25000	~25000	~25000
V_Dee, peak_ [kV]	2.8	6.3	10

A synchrocyclotron operating at fixed beam energy often uses a rotating-vane capacitor (condenser) coupled to a self-excited, broadband RF amplifier to generate the time-varying frequency sweep needed to accelerate beam bunches from injection through extraction (46). Although the sweep is not typically linear in time (depending on the field index, as well as injection and extraction considerations), it can readily be programmed into the mechanical design of the capacitor vanes (47).

Because the high and low limits for the frequency sweep in an iron-free cyclotron are different for each desired final beam energy, and because the total frequency range across all energies is nearly twice that achievable by a conventional single rotating capacitor, we developed a different RF control strategy. The chief constraint on this approach was to remain competitive with existing machines. The iron-free cyclotron must be capable of sweeping each of its frequency ranges at a 1 kHz repetition rate.

**Figure 6:**
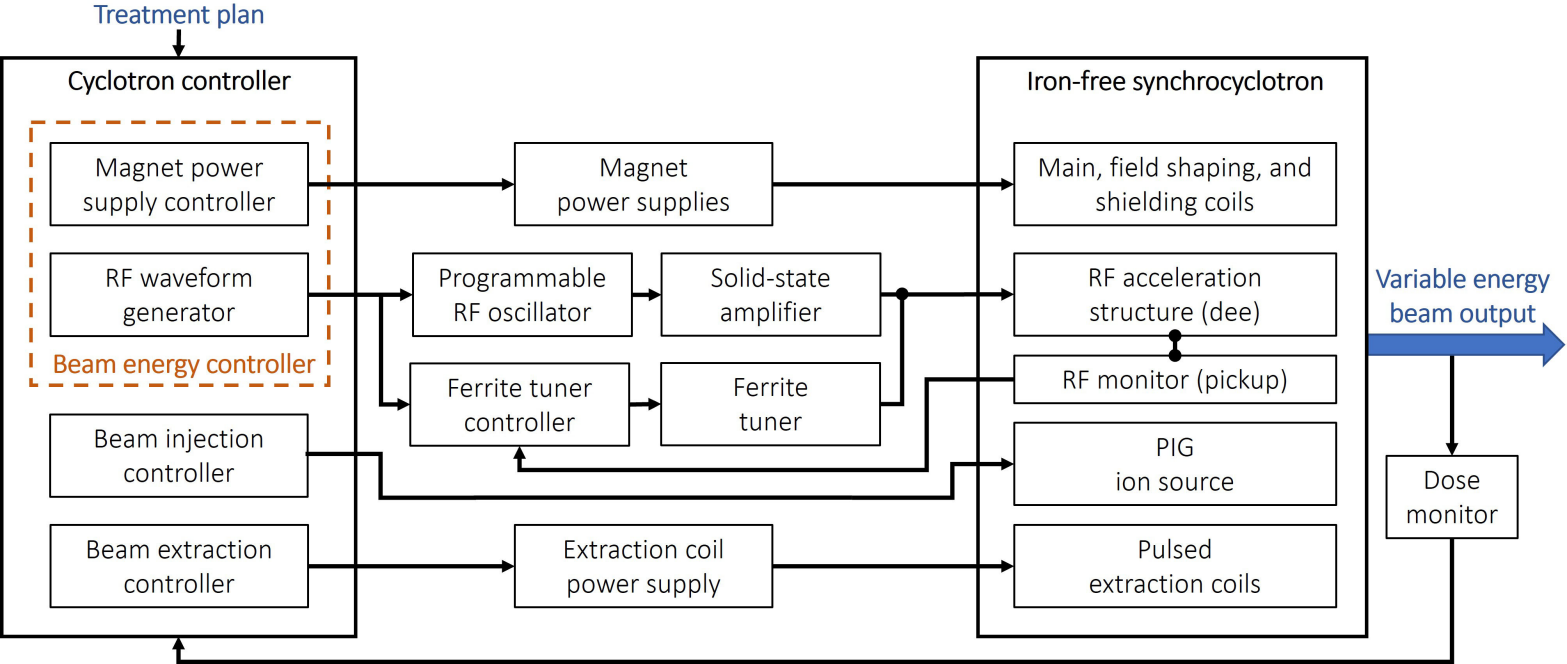
Simplified block diagram of the iron-free cyclotron control scheme.

High-power, solid-state RF technology has progressed rapidly since the development of the rotating-vane capacitor and has reached a state where it is applicable to the RF control systems for medical cyclotrons (48). In **Figure 6** we present a sketch of the proposed RF control scheme. The energy-dependent frequency sweep profile will be generated by a computer-programmable low-level RF oscillator coupled to a solid-state, broadband RF amplifier and at least one magnetically-biased fast ferrite tuner. The bias field on the ferrite tuner will be servo loop controlled with a phase detector and power amplifier arrangement to automatically tune the resonator to the imposed drive frequency. The chief function of the ferrite tuner is to maintain a high Quality Factor (Q) in the RF circuit to minimize the required gain in the RF amplifier.

Perpendicularly-biased ferrite tuners are a reasonably mature technology with broad application to high-energy particle accelerators (45). Even so, both the very broad tuning range – from 34.5 MHz to 75.9 MHz –to cover the iron-free cyclotron’s full energy output range, and the proposed 1 kHz repetition rate are somewhat beyond present state-of-the-art and will require further development to achieve. Based on recent advancements in microwave ferrite materials and recent advances in biased ferrite tuners, we believe this goal is attainable.

The proposed design features a ½-wave microwave-ferrite-loaded and liquid-filled resonator cavity, perpendicularly biased by external, solenoidal magnetic field coils. The external magnet will be enclosed in a general-purpose power transformer ferrite yoke to provide a magnetic field return path to limit the power required to drive the tuner to roughly 1.2 kW. Preliminary calculations for an aluminum-doped garnet ferrite-filled cavity show that the proposed frequency range is easily achievable with an overall minimum cavity Q of approximately 1500. These calculations also indicate an acceptable power loading of roughly 3 kW in the liquid-cooled ferrite to achieve the proposed 10 kV acceleration voltage across the dees. Thus, we estimate the total required RF power to be approximately 10 kW. The ferrite-loaded cavity will be placed outside the cryostat, as close as possible to the dee. The compensation coils of the cyclotron and the yoke of the tuner will effectively shield the ferrites from the cyclotron field.

**Figure 7:**
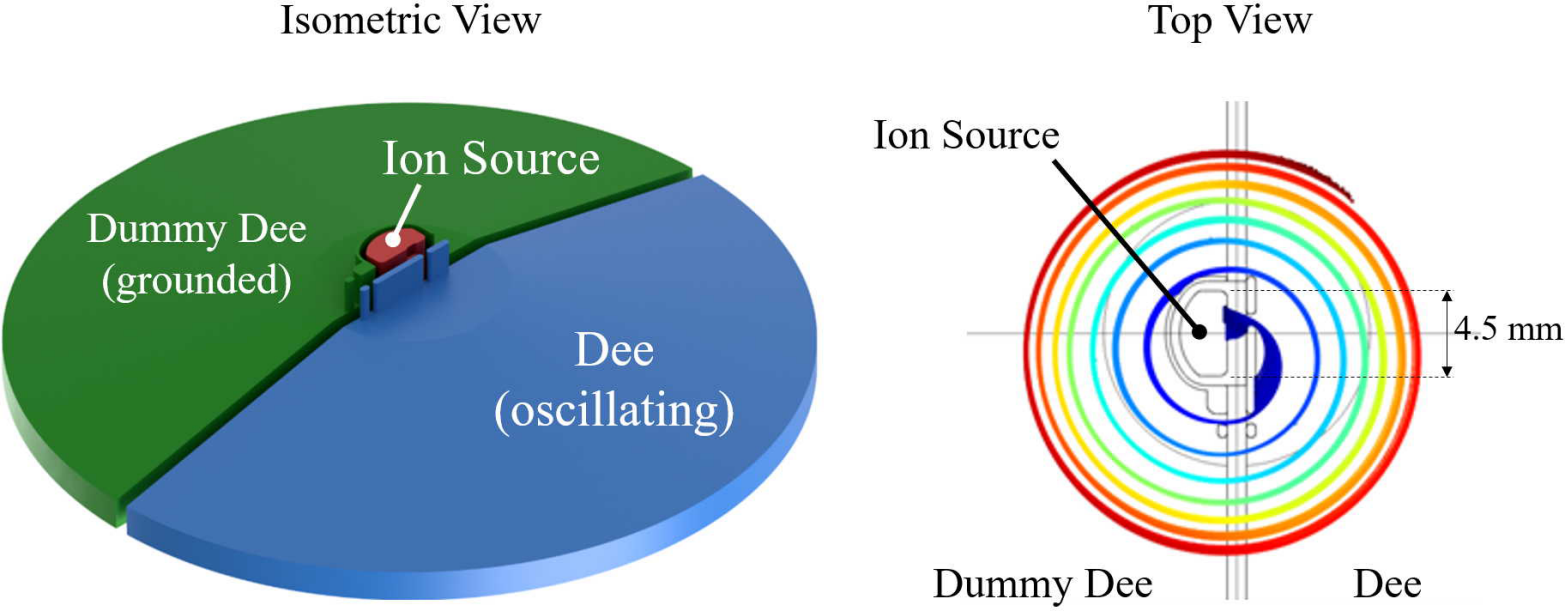
Left: isometric view of the central region model for field calculation and beam dynamics. The ion source can be biased up to 10 kV to compensate for space charge effects. Right: top view of the central region with beam trajectories showing a well-centered beam and phase selection.

The central region of the cyclotron contains the ion source and elements that shape the initial beam. To avoid high heat loads on the cryostat and machine activation from beam lost later during the acceleration cycle, we perform phase selection here (i.e., using collimators, we remove particles that would not be appropriately accelerated). We show the central region in **Figure 7**, left.

### Beam dynamics

To demonstrate the feasibility of the iron-free cyclotron design, we performed a simulation study using the Multiphysics software Comsol (49) and the beam dynamics code OPAL (50).

In this study, the three exemplary final energies listed in **Table 1** were simulated from the ion source to the extraction point. With the requirement of 500 nA after extraction, and assuming a 35% extraction efficiency, a 35% capture efficiency in the central region, and 6 µs capture time every 1 ms (1 kHz repetition rate), we must consider a peak current (or average current from the ion source) of about 680 µA. From there on, the increasing beam energy makes space charge (Coulomb interactions of individual particles in the bunches) less and less important. A study performed by IBA for the S2C2 with similar beam currents showed that space charge led to an overall decrease of current of about 5% after extraction, with no noticeable change in beam quality. Hence, we neglect space charge, knowing that the ion source can deliver significantly more current than we require. With this in mind, we also neglected plasma effects in the source in this conceptual study. We have plans for a small simulation campaign to better understand extraction from the source and its effect on beam quality and current in the future.

We calculated particle trajectories in the central region with COMSOL, using a constant magnetic field and a time-varying electric field, also calculated in COMSOL. The Penning Discharge-type ion source is 4.5 mm in diameter. We place posts to intercept particles that arrive at the wrong RF-phase (i.e., when the accelerating fields are weak) to select only particles within a small phase window suitable for acceleration. We show a typical set of trajectories in **Figure 7**, right. By adjusting the RF frequency, magnetic field, and peak voltage on the dee, we can generate collinear trajectories for all final energies listed in **Table 1**.

To simulate the acceleration to the nominal extraction energy, we generate a 3D field map of the entire acceleration electrode system (also known as *dee* and *dummy-dee*) and use it with the magnetic field to track particles in OPAL.

**Figure 8:**
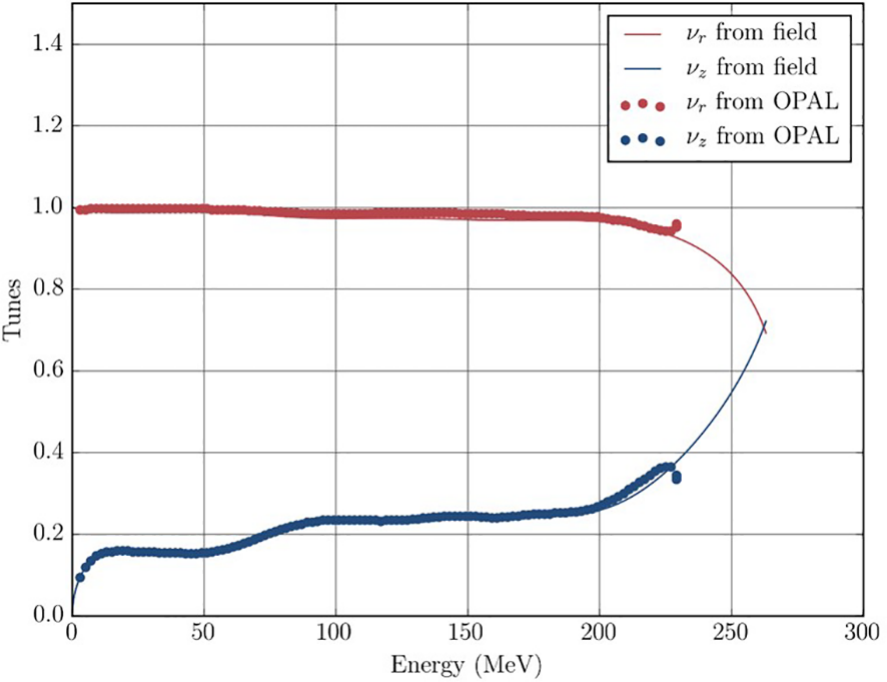
Simulated and calculated tunes during a full acceleration cycle. The up- and downturns at the final energy is the “locking-in” of the resonance caused by the peeler-regenerator system.

As a first step, we performed single and dual particle tracking using the OPAL code to demonstrate the validity of the magnetic field map and 3D electric field map for the dees. We plot the resulting tunes $\nu_{r}$ and $\nu_{z}$ (see Equation 1) using the built-in tune calculation in OPAL, together with the theoretical tunes obtained from analyzing the magnetic field map in **Figure 8**. We observed good agreement. Here, the simulated tunes include the peeler and regenerator fields (see below), which leads to the up- and down-turns of the tunes at the end.

**Figure 9:**
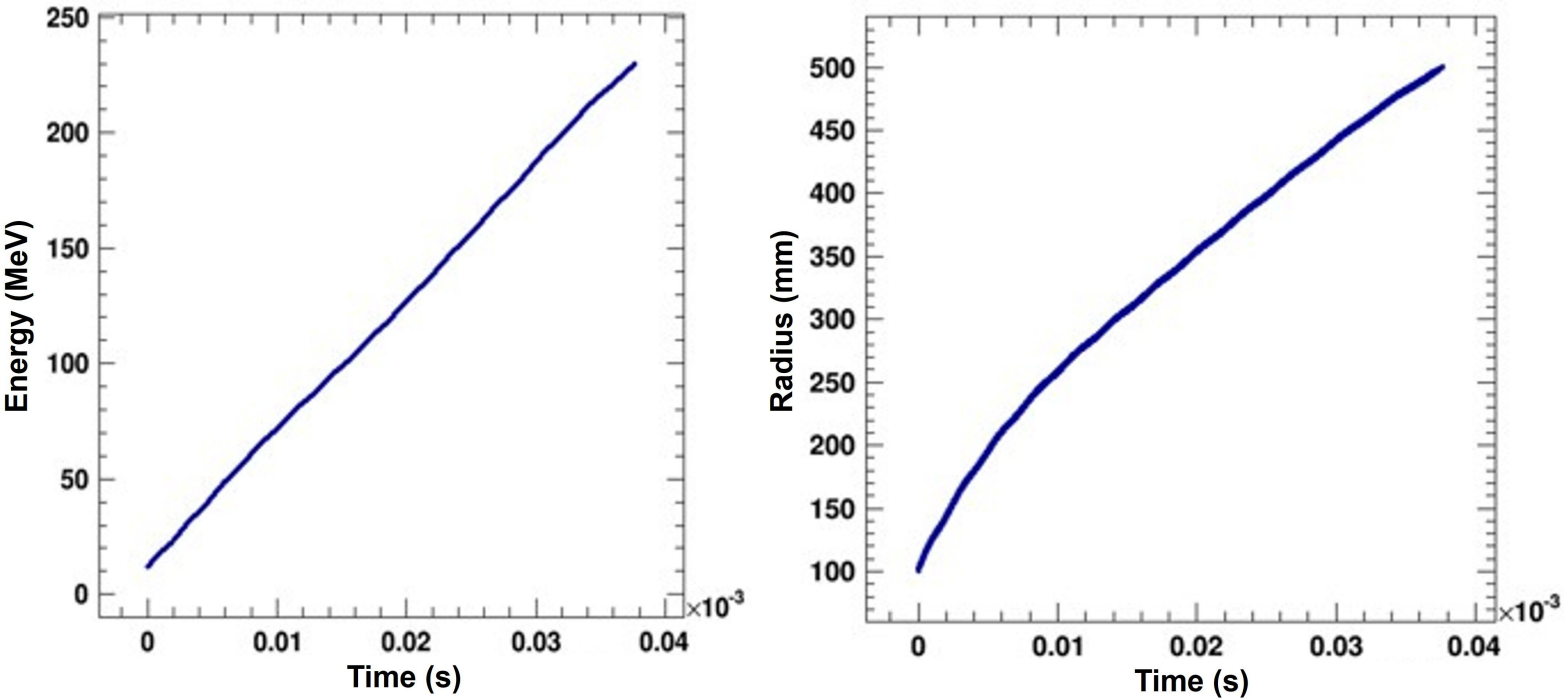
Energy and radius of the bunch centroid as function of time-of-flight. We show the 230 MeV case, which is representative of all cases.

Using the techniques described in the previous section, we then used a Gaussian initial beam (σ = 2 mm) starting at R = 100 mm with 10000 simulation particles to demonstrate acceleration of a bunch to the final energy. This beam is idealized and initially does not have emittance or energy spread. The size was very conservatively estimated from the worst case observed during the central region tracking. We observed mild beam growth to σ = 3 mm in the radial direction and negligible beam growth in the vertical direction for the full acceleration cycle. See **Figure 9** for energy gain vs. time of flight and reference radius vs. time of flight.

Having demonstrated the acceleration up to the final energy, we stopped the beam at R = 45 cm and only tracked bunches from R = 45 cm to R = 50 cm (extraction radius) for the extraction simulations described below. We did this to conserve computational resources. In the next iteration of simulations, we will use the actual particle distribution from the central region studies and track them through the cyclotron.

**Figure 10:**
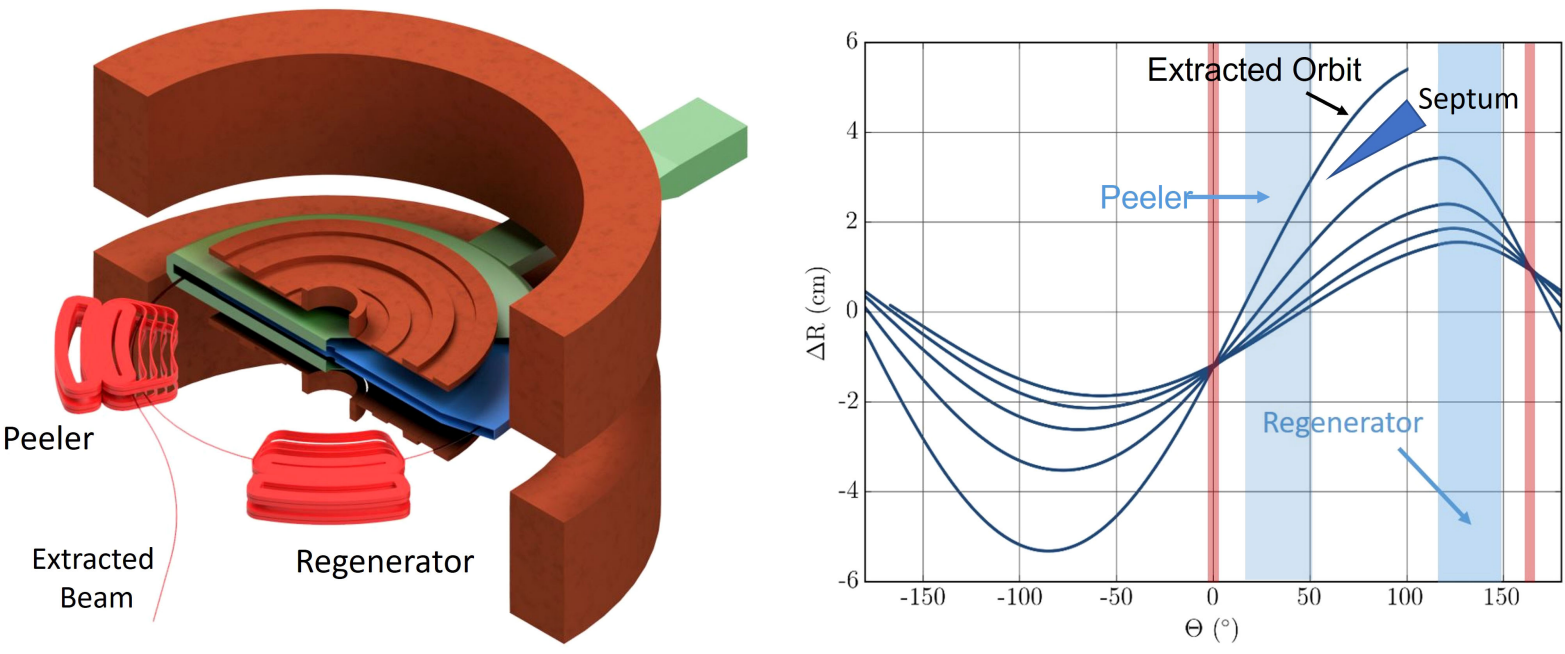
Left: Isometric view of the extraction coils (cf. text) together with the main- and shaping coils, and the acceleration system. Note that we cut open the dummy-dee (green) to expose the dee (blue) for visibility. We also added a schematic of the beam to guide the eye. Right: Beam excursion from the circular reference orbit vs. azimuth during the final five turns with nodes (red) and azimuthal positions of peeler and regenerator coils indicated. Note that the peeler field can be very weak and sometimes omitted altogether as the falloff of the main magnetic field introduces a similar action.

When the beam has reached its nominal energy, we employ *resonant extraction* (51–53), using the ν_r_ = 2/2 resonance^1^. We initiate resonant extraction using two pairs of field perturbation coils located near the outer radius of the beam chamber, also known as a *peeler-regenerator* system. **Figure 10**, left, shows the regenerator coil and its position relative to the edge of the cyclotron. In the absence of iron, the return flux would strongly influence the main acceleration. We generate a set of numerically optimized compensation coils around the peeler and regenerator coils to compensate for this (details about the extraction coils can be found in **Appendix C**). The fields of the peeler and regenerator coils are, again, calculated as 3D field maps and superimposed onto the main magnetic field during tracking. **Figure 10**, right, shows the effect of these two field perturbations on the last five orbits. The beam sizes and energy spread for our set of final energies are listed in **Table 2**.

**Table 2:** Beam sizes and energy spread of the bunch before entering the electrostatic septum and extraction channel (guiding the beam outside through the main coils and cryostat).

Design energy (MeV)	70	150	230
σ_r_ (mm)	0.81	0.67	0.63
σ_z_ (mm)	0.55	0.66	0.71
ΔE (1-sigma) (MeV)	0.01	0.02	0.03

All values are one root-mean-square (RMS). Emittances are normalized to the beam velocity.

With this system, we achieve a turn-separation similar to other proton therapy cyclotrons (16) that allows beam extraction with an efficiency between 35% and 50% of the accelerated current, but at all energies.

### Extraction of the beam

At this current conceptual design stage, we have not performed full tracking simulations for the beam beyond passing through the peeler-regenerator system and demonstrating sufficient turn separation using iron-free coils to excite the resonance.

Typical extraction systems include further magnetic channels and gradient correctors to shape the beam as it is guided out of the cyclotron through a vacuum pipe passing in between the main coils. Optionally, an electrostatic septum can be inserted between the second-to-last and last turn (see **Figure 10**). To push the beam outwards and to increase the turn separation even more. Having such a device also permits a degree of tuning capability through the applied voltage, which must be scaled in accordance with the main magnetic field.

As mentioned above, typical efficiencies for extraction are between 35% and 50%.

To demonstrate that there are no showstoppers, we have shown that beam can be further manipulated with such an electrostatic septum for individual cases during the design process for multiparticle simulations (54) and that beam can be guided outside the cyclotron in single-particle tracking. Multiparticle simulations of a full extraction channel will be part of a future study.

### Machine activation and heat load on the cold mass

A critical issue to consider in the iron-free cyclotron design is that of radiation generated internally in the cyclotron due to beam losses during acceleration and extraction, and the effect it has on machine access for maintenance and heat load to the coils and cold mass.

While we have not performed a nuclear analysis of internal radiation sources for the iron-free cyclotron concept, we have previously performed detailed analyses using a Monte Carlo transport code for another superconducting cyclotron (with iron yoke) designed for 250 MeV protons at 1 mA beam current, and validated it against other analyses performed for the IBA S2C2 Proteus One superconducting cyclotron. This work is reported in detail in Ref (55). The results indicated that the total nuclear radiation heating power inside the superconducting coils is of the order of 4 W peak during the treatment cycle, which would result in a few mK local temperature rise. This is not an issue for the operation of the cyclotron. We note that, as the losses leading to this radiation are primarily during extraction, the iron yoke does not provide shielding for the superconducting coils in this case, and the results are largely applicable to the iron-free design as well. We also note that the IBA S2C2 has been operating commercially for several years with no negative impact from internal nuclear radiation.

Finally, there is room to further reduce activation of subsystems and heat load on the cold mass by installing graphite collimators between the last circulating turns and the extracted beam.

## Conclusion

Hadron and ion beam radiotherapy is the preferred treatment method for many pediatric cancers. Over the past decade, its use has been rapidly expanding to a wider range of ages and cancer types (56). Until now, the size, the capital- and operating cost, and the complexity of ion beam radiotherapy accelerators, however, have been recognized as a significant impediment to expanding the use of ion beam delivery. The report from the 2013 workshop on Ion Beam Therapy outlined the R&D needed to achieve these goals, including a significant effort required to improve accelerator technology (57). This report explains that only slow-cycling synchrotrons are presently used for treatment with ions heavier than protons. Additional technical discussion of concepts needed to make ion beam therapy “smaller, lighter, and cheaper” is given in the book chapter by Cameron and Schreuder (58), which explains how the application of superconductor technology to cyclotrons can achieve these goals. Indeed, significant efforts are being pursued to develop more compact hadron therapy solutions that leverage superconductivity (59). Developing a highly compact cyclotron to accelerate protons and heavier ions up to carbon while allowing for variable energy extraction combines the advantages of previous systems with a readiness for future treatment modalities (58).

If proven, iron-free synchrocyclotrons could provide beam energy variation without a degrader and could potentially be used with various accelerated particles in a single device. While we still need to demonstrate the concept’s full potential, we already see that, compared with current cyclotrons, the iron-free design would provide similar flexibility and advantages for patient treatment delivery, including a modified pencil beam scanning technique with comparable beam position accuracy and delivery time.

The most significant advantage resides in the potential of this type of cyclotron to provide FLASH treatment by delivering consistently high beam currents across the full energy range, achieving a projected significant reduction in treatment duration (duration of a dose fraction and number of fractions). This cyclotron could be the ultimate optimization for FLASH treatment delivery, providing the highest dose rate, the sharpest beam, and the best energy resolution at any energy, giving it the potential to broaden the scope of flash treatment, particularly for the irradiation of shallow tumors. Elimination of the energy degrader also reduces system complexity and cost.

## Figures and Tables

**Figure 11:**
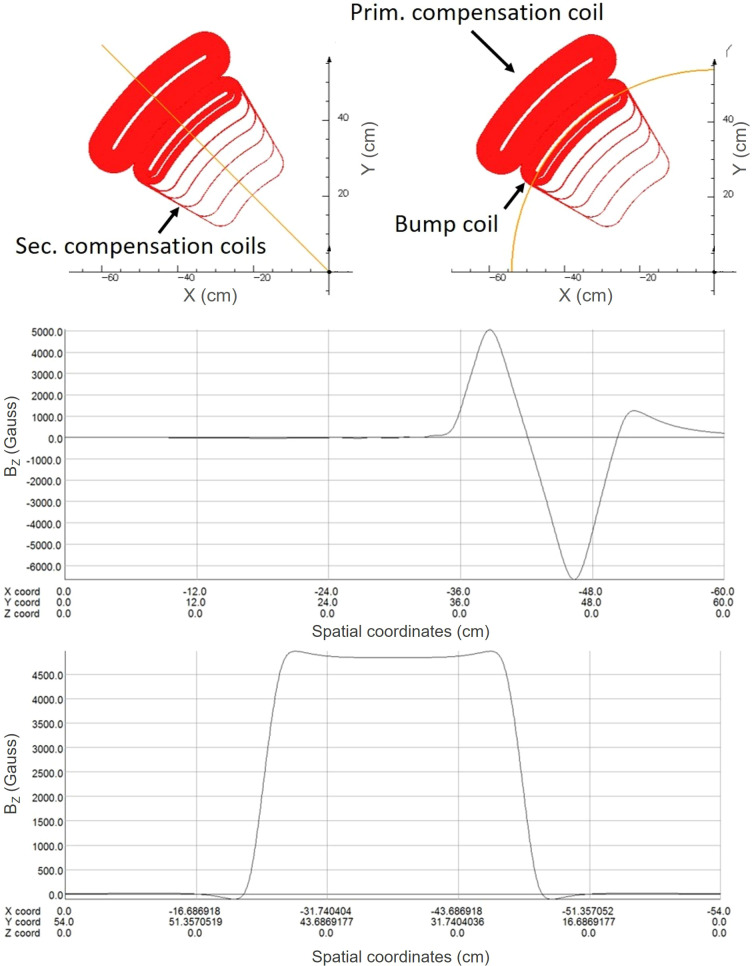
Top view of a regenerator coil with associated vertical magnetic field component (Bz) along two different lines: A line along the radius passing through the middle of the coils (upper field plot), and a line along a circle passing through the center of the main bump coil (lower field plot). See **Figure 10** for a schematic placement of the regenerator coil in the overall system.

## Data Availability

The raw data supporting the conclusions of this article will be made available by the authors, without undue reservation.

## Footnotes

1 Such a resonance happens when the particle performs one full radial oscillation about the circular reference orbit per turn. The 2/2 refers to the fact that two second-order perturbations in the magnetic field lead to this particle behavior. During each revolution, the particle goes more eccentric, creating room for extraction elements between turns. See **Appendix C** for a more detailed explanation.

## Appendix A: basic relativistic cyclotron equations and choice of cyclotron type:

In all cyclotrons, as the beam energy increases, the relativistic change of inertia leads to particles slowly losing synchronicity with the accelerating, oscillating electric fields created by radio-frequency (RF) cavities. The governing equations for gyration radius *r* and revolution frequency *ω* of the particle are:

(5)
$$r=\;\frac{\gamma m_{0}\beta c}{qB}$$

(6)
$$\omega =\;\frac{qB}{\gamma m_{0}}$$

With *ß* and *γ* the relativistic factors, *c* the speed of light, *q* and *m_0_* the charge and mass of the particle, respectively, and *B* the magnetic field. Two cyclotron concepts circumvent this issue: The isochronous cyclotron, where the magnetic field is adjusted to increase with radius to keep ω constant, and the synchrocyclotron, where the frequency of the cavities is changed as particles gain energy. In this design, we chose the synchrocyclotron due to its rotational symmetry, which lends itself more easily to an iron-free design.

## Appendix B: beam dynamics simulation codes:

Here we provide a brief overview of the design and simulation codes we used for this project. The software can be grouped into three categories:

- Finite Elements Analysis/Methods (FEA/FEM) codes to calculate electromagnetic fields, analyze structural integrity, and analyze thermal properties. We used OPERA (60) and COMSOL (49) for this purpose. Both have similar capabilities, and for magnetic and electric field calculations, as well as particle tracing, we corroborated results from one with the other. We used OPERA for the structural analysis. We used COMSOL for the detailed central region design.
- Beam dynamics codes. We used OPERA and COMSOL for single particle tracing of short tracks in the central region and during extraction. Here, a fast turnaround with immediate visual feedback was helpful in the design process. For longer particle tracing with many particles (typically 10000) per bunch, we used the particle-in-cell (PIC) code OPAL (50). OPAL is a 3D particle tracking code with a dedicated “cyclotron” module. OPAL is highly parallel and can be deployed on clusters. We used the Engaging cluster at MIT to run simulations of the acceleration from the center to the final energy (around 10000 revolutions, or “turns” inside the cyclotron) as well as of the extraction process. Due to the low beam currents typically accelerated by synchrocyclotrons, we neglected space charge during the simulation.
- Design and utility codes. To generate the extraction coils from an analytical theory (see **Appendix C**) and perform preliminary beam tracking, we used our own code written in Python 3.7 (61) utilizing mathematical methods provided by the NumPy and SciPy modules. We also used Python for data preparation, analysis, and visualization.

## Appendix C: details of the extraction coils:

The intent of the extraction coils is to provide a highly localized perturbation to the magnetic field structure while minimizing field perturbations elsewhere. The local field perturbation rises (regenerator) or falls (peeler) linearly with radius and causes the center of the beam orbits to precess away from the center of the cyclotron, opening increasingly wider gaps between orbits. When the gap near the extraction location is large enough, an electrostatic deflector will be used to bring the beam out from the cyclotron through a dedicated beam extraction channel. This process is called *regenerative extraction* using a peeler-regenerator system and is described in more detail elsewhere (51, 52, 62, 63). Based on these references, we developed a matrix-based particle tracking code in Python to estimate the effects of local perturbations. This allows us to determine an optimal field gradient for given radial and azimuthal positions of the coils and their angular width.

In order to achieve the needed local field modifications for extraction without introducing a harmonic perturbation during the main acceleration phase, we place additional coils around the main bump coil. We wrote a Python program that uses numerical optimization routines from the SciPy module to achieve the best possible compensation. Here, the desired gradient in Gauss/cm can be specified together with an azimuthal width and a start and end radius for the linear perturbation. As an example, we show the magnetic field in the vertical direction along a radial line passing the coil in the middle and along an azimuthal line in **Figure 11**.

The amplitude of field perturbation needed for extraction varies directly with the extracted beam energy. That is, in an ideal case, the extraction coils would be connected in series with, and operate at, the same current as the field shaping coils, but, if desired, they can be on a separate circuit for added tuning capabilities.

The design of the extraction coils is highly constrained. Foremost, to achieve sharp magnetic field boundaries, the coils are required to operate at as high an overall current density as feasible. This requirement generally precludes distributed cooling throughout the coil section, placing a high priority on the use of High-Temperature Superconductors (HTS) that can be conduction-cooled to higher than the nominal operating temperature from the rest of the superconducting coil sets. Fine filamentary conductors are preferred to limit self-heating in the presence of changing magnetic fields and currents. A leading HTS candidate for use in the extraction coils is reinforced, melt-textured Bi_2_Sr_2_CaCu_2_O_8_ (Bi-2212) operating at temperatures below roughly 25 K peak temperature (64, 65), or the exfoliated and re-stacked fine filamentary REBCO conductors under development by BTG (66).
